# The Canadian 2014 porcine epidemic diarrhoea virus outbreak: Important risk factors that were not considered in the epidemiological investigation could change the conclusions

**DOI:** 10.1111/tbed.13496

**Published:** 2020-02-16

**Authors:** Louis E. Russell, Javier Polo, David Meeker

**Affiliations:** ^1^ APC LLC Ankeny IA USA; ^2^ APC Europe, S.L.U. Granollers Barcelona Spain; ^3^ North American Renderers Association Alexandria VA USA

**Keywords:** Canada, epidemiology, feed, feed ingredients, porcine epidemic diarrhoea virus, risk factors, spray‐dried porcine plasma

## Abstract

The introduction and spread of porcine epidemic diarrhoea virus (PEDV) in North America resulted in significant death loss in the swine industry. As the industry learned how to manage this disease, many new risks were identified, including the potential for feed and feed ingredients to become contaminated and spread PEDV. In addition, biosecurity practices were reevaluated and strengthened throughout the industry. At the time of the outbreak epidemiologists did not understand, as well as they are understood today, all the risk factors that contribute to the spread of PEDV. As a result, the epidemiological investigations into the 2014 PEDV outbreak in eastern Canada may not have investigated all risk factors as thoroughly as they would be investigated today. In retrospect, many of the Bradford Hill criteria used to determine causation were not fulfilled. This review identifies risk factors that were not included in the 2014 epidemiology. If these risk factors were included in the epidemiology, the conclusions and determination of causation may have been different.

## 1 INTRODUCTION

Epidemiological investigations typically examine associations between exposure variables and health outcome. The “Bradford Hill criterion” provides a framework for assessing the causal nature of an observed association (Hill, 1965). The process of causal inference is complex and can be subjective. Causation becomes unlikely as non‐compliance with the Bradford Hill criterion increases.

Briefly, the criterion includes:

- *Strength of the association*. The stronger the association between a risk factor and outcome, the more likely the relationship is to be causal.
- *Consistency of findings*. The response to the risk factor should be consistent in different populations, different places, circumstances and times.
- *Specificity of the association*. There must be a direct relationship between cause and outcome.
- *Temporal sequence of association*. Exposure to the risk factor must precede the outcome.
- *Biological gradient*. Increasing exposure to the variable should result in increasing the rate of the outcome.
- *Biological plausibility*. Is there a potential biological mechanism?
- *Coherence*. Does the relationship agree with the current knowledge of the natural history/biology of the disease?
- *Experiment*. Does the removal of the exposure alter the frequency of the outcome?

Spray‐dried animal plasma (SDAP) is a feed ingredient commonly used in diets of young animals including pigs, calves and poultry. Typically, SDAP is manufactured from blood collected from pigs (SDPP) or cattle (SDBP) at federally inspected abattoirs. When SDAP is included in starter diets, pigs consume more feed, grow faster, diarrhoea and mortality are reduced and health is improved (Coffey & Cromwell, 2001; Dewey, Johnston, Gould, & Whiting, 2006; Pérez‐Bosque, Polo, & Torrallardona, 2016; Remus et al., 2013; Torrallardona, 2010; van Dijk, Everts, Nabuurs, Margry, & Beynen, 2001). Dietary SDAP has been shown to reduce intestinal inflammation, improve intestinal barrier function, reduce gut leakage, increase nutrient absorption, restore defensin production and normalize balance of intestinal microbiome (Campbell, Crenshaw, Gonzalez‐Esquerra, & Polo, 2019; Campbell, Polo, Russell, & Crenshaw, 2010; Moretó, Miró, Amat, Polo, & Pérez‐Bosque, 2017; Moretó & Pérez‐Bosque, 2009; Moretó et al., 2019; Pérez‐Bosque et al., 2010; Tran et al., 2018). In mice challenged with inhaled LPS, dietary SDPP reduced inflammatory cytokine production in lung tissue, reduced shedding of lymphocytes in BALF fluid and reduced other inflammatory markers (Maijó et al., 2012b, 2012a). Inbred mice subjected to transport stress, dietary SDPP has been shown to improve embryo implantation and survival, improve foetal growth and reduce uterine inflammation (Liu et al., 2018; Song et al., 2015). Normal intestinal and cerebral inflammation associated with ageing is reduced when SDPP is included in the diet (Miró et al., 2017; Moretó et al., 2018). Recognizing the uniqueness of SDAP, the American Society of Animal Science identified SDAP among the most important discoveries in swine nutrition in the past century (Cromwell, 2009).

The first case of porcine epidemic diarrhoea virus (PEDV) in the United States was reported in April 2013 (AASV, 2013). The virus spread rapidly and within weeks PEDV was reported in 12 states (Niederwerder & Hesse, 2017). Soon after the initial outbreaks, some veterinarians began to suspect that feed may have been involved in the spread of PEDV. Because of positive PEDV PCR test results, many began to suspect SDPP as a potential contaminated feed ingredient for PEDV cases related to feed (Bowman, Krogwold, Price, Davis, & Moeller, 2015; Byrne, 2014; Newman & Gee, 2014; Nugent, 2015; Sampedro et al., 2014).

Canada reported the first confirmed case of PEDV on 22 January 2014 in Ontario (Ojkic et al., 2015). Following the initial outbreak, federal and provincial officials, university researchers and private veterinarians initiated an epidemiological investigation to determine how PEDV was introduced into Canada (Aubry, Thompson, Pasma, Furness, & Tataryn, 2017; Kochhar, 2014; Pasick et al., 2014; Pasma, Furness, Alves, & Aubry, 2016). Collectively, the conclusion of the Canadian investigation was summarized:

The weight of evidence gathered during an outbreak of porcine epidemic diarrhea (PED) in Canada in January 2014 supports an association with feed containing spray dried porcine plasma contaminated with the virus. (Aubry et al., 2017)

When used correctly, SDPP significantly improves swine production efficiency. However, there is confusion concerning the relative risk associated with using this ingredient. The Canadian epidemiology from the 2014 PEDV outbreak is commonly cited when the risk of feeding SDPP is discussed (Davies, 2015; USDA APHIS, 2019). Since the initial outbreak, much has been learned about PEDV transmission resulting in improved biosecurity procedures (ANAC, 2018; Cochrane et al., 2016; Kim, Yang, Goyal, Cheeran, & Torremorell, 2017; National Pork Board. PEDV Resources, 2015a). In retrospect, the Canadian epidemiology did not investigate many PEDV risk factors as thoroughly as they would today because, at the time, PEDV risk factors were not understood as well. In addition, several of the Bradford Hill criteria were not fulfilled making it more difficult to determine causation. If these risk factors had been included in the investigation, it is possible that the conclusions may have been different which could also change current perceptions of the risk associated with feeding SDPP. This review is a retrospective evaluation of the epidemiology from the Canadian PEDV outbreak and highlights how the conclusions may have been different if the additional information had been considered.

## 2 RETROSPECTIVE REVIEW OF EPIDEMIOLOGY FROM 2014 CANADIAN PEDV OUTBREAK

### 2.1 The Index case may not represent the first introduction of PEDV into eastern Canada

**Figure 1:**
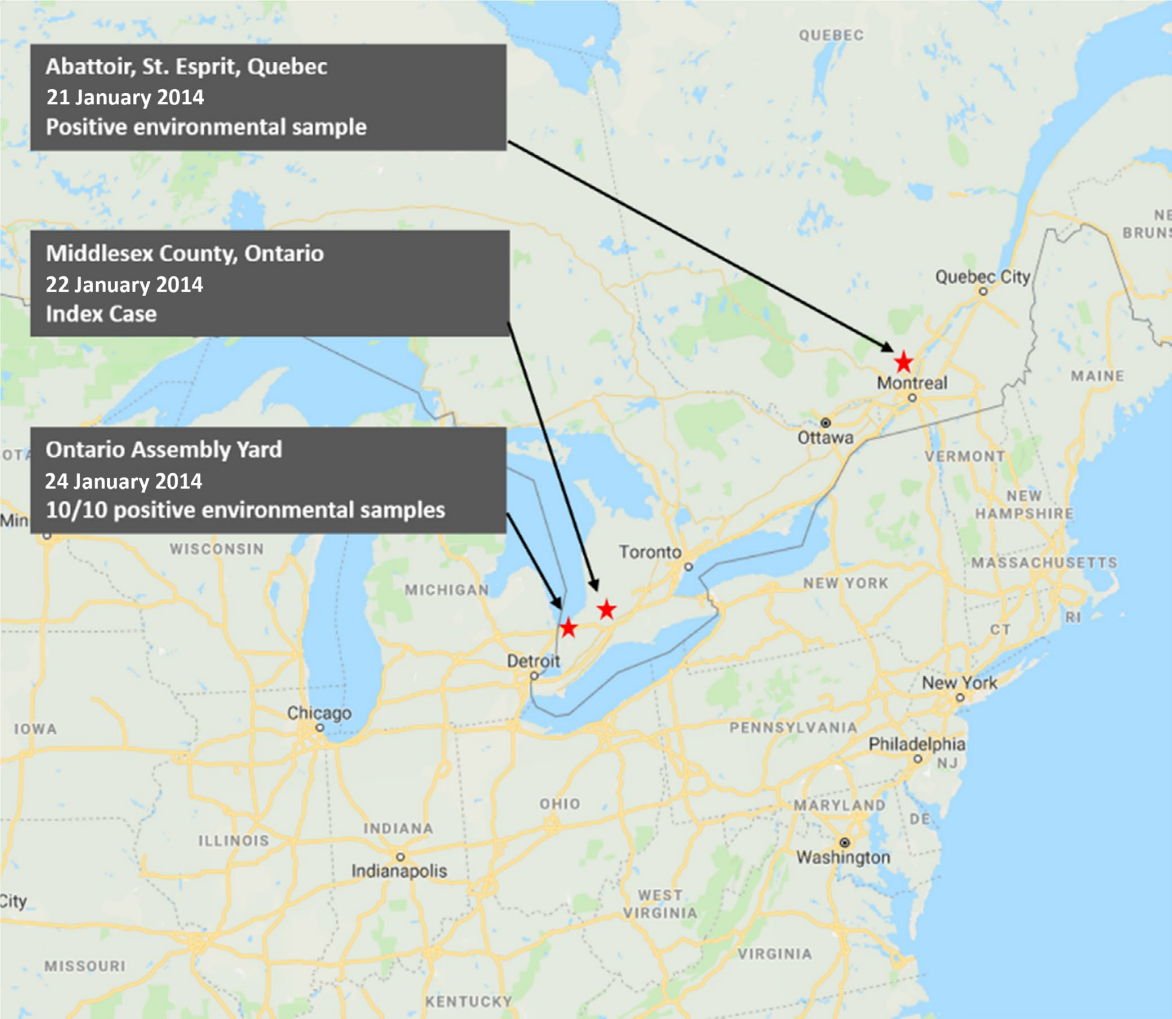
Timeline and geographic distribution of porcine epidemic diarrhoea virus detection in eastern Canada

A review of the timeline associated with the Index case and reports of PEDV‐positive environmental samples suggest that PEDV was present in multiple locations in Quebec and Ontario prior to the Index case. The Index case may not represent the first introduction of PEDV into eastern Canada (Figure 1).

On January 21, 2014 a pork slaughter plant in Saint‐Esprit, Quebec reported PEDV positive environmental samples. (Bedard, 2014; Mann, 2014)

On January 22, 2014 the Index case was confirmed by CFIA on a pig farm in Middlesex, Ontario. (Ojkic et al., 2015)

On January 24, 2014 the CFIA confirmed PEDV was detected in all 10 environmental samples from an Ontario assembly yard. (Pasma et al., 2016)

Typically, SDPP is included in pig nursery feeds and is not included in finisher diets (KSU Premix and Diet Recommendations, 2019). Detection of PEDV in the Quebec abattoir suggests that market hogs were infected with PEDV. It would not be expected for SDPP to be the source of the PEDV found at an abattoir.

Two days after identifying the Index case, epidemiologists reported significant PEDV contamination at an Ontario assembly yard. Prior to collecting these samples, regular environmental monitoring was not reported at the assembly yard. Therefore, it is not possible to determine when PEDV was first introduced into this site. It is possible that the PEDV was present at the assembly yard before the Index case.

Based on the timeline and location of environmental contamination, it is possible that the 2014 Index case does not represent the introduction of PEDV in eastern Canada. If PEDV had contaminated a common site prior to the Index case being reported, it becomes more difficult to differentiate the origin of virus in subsequent outbreaks.

#### 2.1.1 If the Index case did not introduce PEDV into Eastern Canada, how could PEDV have been introduced? Truck and animal movement

Trucks returning from contaminated abattoirs represent a significant risk of being contaminated with PEDV (Boniotti et al., 2018; Lowe, 2014; Lowe et al., 2014; Machado et al., 2019; Sasaki et al., 2016; Tousignant, 2015). Current truck wash protocols to prevent the spread of PEDV are extensive and include multiple steps (ANAC, 2018; National Pork Board, 2015b):

1. Removal of all manure and bedding.
2. Soaking with soap and/or degreaser.
3. Pressure washing with hot water is most effective versus cold water wash.
4. Disinfecting by foaming with an appropriate disinfectant.
5. Drying, including heat and/or fans.

**Figure 2:**
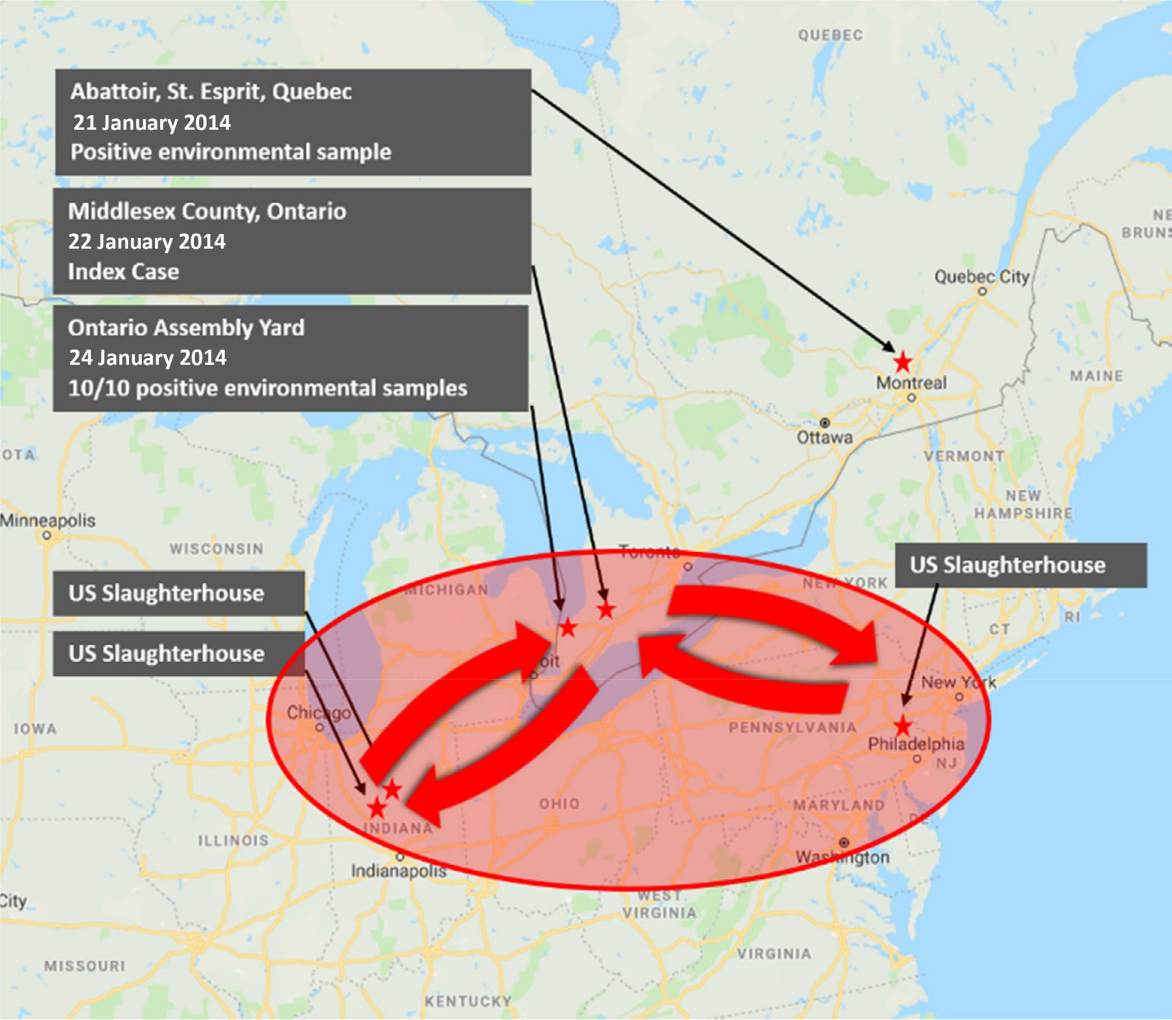
Market hogs were regularly transported from SW Ontario to US abattoirs during the time of porcine epidemic diarrhoea virus outbreak

At the time of the PEDV outbreak, trucks were regularly transporting Canadian pigs to US slaughter plants (Bedard, 2014; Figure 2). In early 2014, Canadian regulations required that trucks returning from US abattoirs directly to Canada only be cleaned of visible manure using a shovel and broom (Table 1).

**Table 1:** Canadian animal health regulations: cleaning of trucks returning from the United States

(5.1) No person shall bring from the United States a conveyance that has been used to transport poultry or porcine unless it has been cleaned and disinfected. (5.2) Subsection (5.1) does not apply to a conveyance a. that has transported Canadian porcine to a slaughtering establishment in the United States where inspection is provided by the Food Safety and Inspection Service of the United States Department of Agriculture and that has returned directly to Canada from that establishment; b. that has not transported porcine other than those mentioned in paragraph (a) while in the United States; c. that is not licensed to transport livestock between locations in the United States; and d. from which as much manure as could be removed with a shovel and broom at an ambient temperature of 20°C has, in fact, been removed.

Note

Health of Animals Regulations (C.R.C., c. 296) Part X. Disinfection. Section 106 (http://laws-lois.justice.gc.ca/eng/regulations/C.R.C.,_c._296/20121214/P1TT3xt3.html).

Comparing current truck washing and disinfection protocols and the CFIA requirements in place in 2014, it is possible that some of the trucks returning to Canada from US abattoirs were inadequately cleaned. Therefore, contaminated trucks could have brought PEDV into Canada. In retrospect, PEDV‐contaminated trucks returning from US abattoirs is a more logical explanation for the extensive environmental contamination found at the Ontario assembly yard and at the Quebec abattoir compared to the alternative explanation that the environmental contamination was a result of PEDV originating from the Index case.

#### 2.1.2 If the Index case did not introduce PEDV into Eastern Canada, how could PEDV have been introduced? Non‐animal feed ingredients

Original research (Dee, Clement, et al., 2014; Dee, Neill, Clement, Christopher‐Hennings, & Nelson, 2014; Dee et al., 2016) and recent review papers (Gordon et al., 2019; Jones, Woodworth, Dritz, & Paulk, 2019; USDA APHIS, 2019) have begun to identify and assess the risk of feed and non‐animal feed ingredient as potential fomites for spreading virus. For example, PEDV has been shown to survive for extended periods of time on conventional and specialty soybean meal (Dee et al., 2015, 2016; Trudeau, Verma, Sampedro, et al., 2017; Trudeau, Verma, Uriolla, et al., 2017). Current biosecurity protocols for sourcing, transport and receiving bulk feed ingredients have been enhanced relative to those in place prior to the introduction of PEDV into North America (AFIA, 2019; ANAC, 2018; National Pork Board, 2015b).

During 2014, Canada imported over a million‐metric ton of soybean meal from the United States (USSEC, 2015). The Canadian epidemiology did not detect PEDV contamination in the feed mill, delivery trucks or other feed ingredients in the feed mill that produced the suspect feed (Pasma et al., 2016). However, other feed manufacturers could have imported soybean meal or other non‐animal feed ingredients that became contaminated with PEDV before or during transportation from the United States or other countries and subsequently incorporated into grower‐finisher feed resulting in PEDV‐infected market hogs. At the time of the Index case, biohazard risk for PEDV in incoming feed ingredients to feed mills were not well understood (Cochrane et al., 2016). Contaminated non‐animal feed ingredients infecting market hogs could be an alternative explanation of a potential source of the PEDV‐positive environmental samples at the Quebec abattoir and the Ontario assembly yard (Bedard, 2014; Mann, 2014). If it had been better understood at the time, the Canadian epidemiology could have investigated the risk of non‐animal feed ingredients more thoroughly.

### 2.2 Canadian epidemiology did not include animal and truck contact with PEDV‐contaminated sites

Epidemiological investigations (Sasaki et al., 2016; Tousignant, 2015) and the use of predictive algorithms (Machado et al., 2019) identified risk factors associated with the spread of PEDV including local pig density, proximity to an infected farm, local pig movements, the number of feed deliveries and environmental factors (vegetation, wind speed, temperature, precipitation and topographical features such as slope). Animal and truck movement through high traffic areas, especially through PEDV‐contaminated areas, significantly increase the risk of spreading PEDV (Lowe et al., 2014; Machado et al., 2019).

In addition to the initial epidemiology (Aubry et al., 2017; Kochhar, 2014; Pasick et al., 2014; Pasma et al., 2016), recent publications describe further examination of the Canadian 2014 PEDV outbreak including a case‐controlled study (Perri, Poljak, Dewey, Harding, & O'Sullivan, 2018) and network analysis (Perri, Poljak, Dewey, Harding, & O'Sullivan, 2019). In these recent papers, a subset including nine of the initial 25 PEDV Case herds were selected and paired with corresponding control herds. After agreeing to participate, the producer was interviewed to collect information on feed deliveries, animal movement, service providers and other potential risk factors. Consistent with the initial reports, the more recent analysis by either methodology (case‐controlled study or network analysis) resulted in the conclusion that the early PEDV outbreak was likely the result of PEDV‐contaminated feed from a single feed supplier. Further, the epidemiologists were unable to detect a link with other risk factors including animal movement, transportation companies or other risk factors (Perri et al., 2018, 2019).

**Figure 3:**
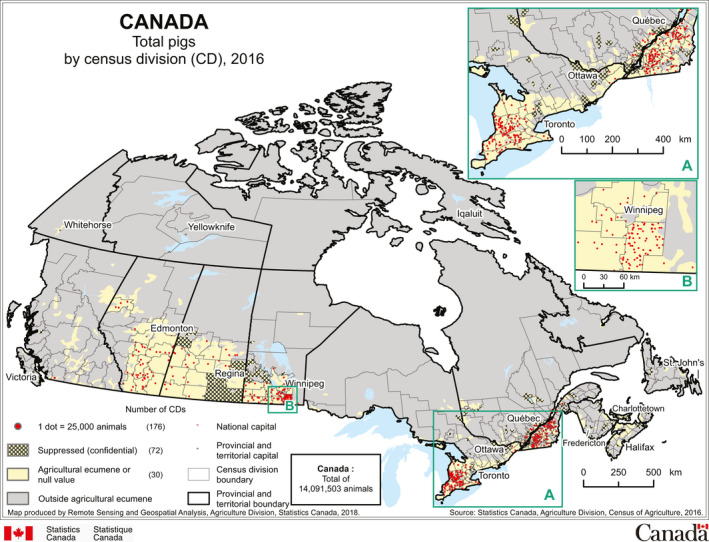
Distribution of pigs within Canada, 2016

The initial PEDV‐infected farms in Ontario were in the pig dense southwest region of the province (Figure 3; Pasma et al., 2016). The contaminated assembly yard was also located in the same region (Figure 1; MacDougald, 2014a). Eight of the initial 25 PEDV cases had contact with the contaminated assembly yard within 2 weeks of the outbreak (Aubry et al., 2017; Pasma et al., 2016). However, it was not possible to investigate this risk because of the difficulty tracing animal movement through multiple sites before reaching a destination (Pasma et al., 2016). This creates the opportunity for exposing animals to pathogens and contaminating transportation equipment (Lowe, 2014; Lowe et al., 2014; Machado et al., 2019). The case–control study and network analysis captured information on the origin and destination of animal movement but did not include information on prior contact of the transportation equipment with other sites (Perri et al., 2018, 2019).

The US swine industry reported PEDV outbreaks before cases were reported in Canada (AASV, 2013; Ojkic et al., 2015). PEDV has been shown to survive on complete feed formulated without animal origin ingredients and non‐animal origin feed ingredients (Dee, Clement, et al., 2014; Dee, Neill, et al., 2014; Dee et al., 2015, 2016; Gebhardt et al., 2018). Biosecurity protocols for ingredient receiving, feed manufacturing and feed delivery have been enhanced since PEDV entered North America (AFIA, 2019; Cochrane et al., 2016; Kim et al., 2017; PIC, 2019). There is a greater risk of feed mill contamination during cold weather due to ice and snow accumulation on trucks (Cochrane et al., 2016). The manufacturer producing the feed suspected of PEDV contamination supplied both Canadian and US producers (L. E. Russell, personal communication, April 8, 2015). The Canadian epidemiology did not include an assessment of the feed biosecurity protocols at the time of their investigation (Aubry et al., 2017; Kochhar, 2014; Pasick et al., 2014; Pasma et al., 2016; Perri et al., 2018, 2019). In addition, the complete feed being investigated was pelleted, a process that has been shown to inactivate PEDV (Cochrane et al., 2017). This is consistent with results of the pig bioassay CFIA conducted where infective PEDV was not detected in the complete feed (Pasick et al., 2014) and supports the potential of a breach in feed production/delivery biosecurity protocol contributing to the spread of PEDV. It is possible that feed biosecurity protocols in place at the time of the Canadian PEDV outbreak were not as rigorous as those recommended today and post‐processing PEDV contamination of the feed or feed delivery equipment delivery truck could have contributed to the PEDV outbreak.

It is unfortunate that the Canadian epidemiology did not investigate contact between the assembly yard, farms included in the initial PEDV outbreak, the feed plant that manufactured the nursery feed for the Index cases and other high traffic sites. The inability to investigate the role of direct contact with the contaminated assembly yard does not preclude its contribution to the spread of PEDV. In retrospect, it is possible that truck traffic, animal movement and feed delivery between the contaminated Ontario assembly yard, Quebec abattoir and other common sites contributed to the PEDV outbreak.

### 2.3 FDA did not detect a breach of good manufacturing practices or infective PEDV in the retained samples of the product being investigated in the Canada PEDV outbreak

Epidemiologists investigating the Canadian PEDV outbreak suggest that a breach in good manufacturing practices (GMP's) could result in PEDV contamination of the SDPP being investigated in the Ontario outbreak (Aubry et al., 2017).

As a result of the CFIA investigation into the potential role of US sourced SDPP in spreading PEDV, the FDA investigated the manufacturer of the suspected SDPP. As part of their investigation, the FDA reviewed manufacturing records of the lot of SDPP investigated by CFIA and did not identify a breach of GMP's or preventive controls (NASDBPP, 2014a).

FDA officials also conducted a pig bioassay on retained samples of the lot of SDPP being investigated by CFIA. The FDA test results confirmed that the retained samples were not contaminated with infective PEDV (NASDBPP, 2014b). It is important to recognize the retained samples were stored in the same warehouse under the same environmental conditions as commercial product, not at room temperature which has been shown to inactivate PEDV (Dee et al., 2015; Pujols & Segalés, 2014).

In the months prior to the Canadian PEDV outbreak, the plasma manufacturer regularly exported SDPP PCR positive for PEDV to Brazil and to Western Canada from the same production facility produced under the same GMP's as the lot of SDPP investigated by CFIA. The amount of SDPP exported during this period was enough to feed 2.5–3.5 million pigs in Brazil and 3.5–4.0 million pigs in Western Canada. Neither region experienced a PEDV outbreak during that time period (Crenshaw, Campbell, Campbell, & Polo, 2014; Crenshaw, Pujols, et al., 2014; NASDBPP, 2014b).

The OIE establishes guidelines to assist government regulators and industry professionals to establish trade policy controlling the introduction and spread of animal diseases. When determining the risk of introducing an animal disease by importing commodities of animal origin, the OIE recommends to first determine if the pathogenic agent is present in the animal tissue from which the product is derived. If the disease agent may be present in or may contaminate the tissue from which the product is derived, the OIE recommends determining if the processing method will inactivate the pathogenic agent (OIE Terrestrial Animal Health Code, Chapter 2.2, updated 2019). The OIE Scientific Commission on Animal Diseases determined that SDPP is not a likely source of infectious virus if good manufacturing procedures are followed (OIE Technical Fact Sheet. Infection with Porcine Epidemic Diarrhea Virus, 2014).

The WHO guidelines for assuring the viral safety of human blood products recommend that the production process include either one or two steps able to inactivate four logs of a non‐enveloped or enveloped virus, respectively (WHO Technical Report, Series No. 924, 2004). The SDPP manufacturing process has been shown to inactivate > 4 logs of both envelope and non‐enveloped viruses (Table 2) and selected pathogenic bacteria (Table 3). In addition, published trials report that pigs fed PCR‐positive SDPP at high levels for extended periods of time did not become infected (Table 4). This confirms that while SDPP may be PCR positive, PCR‐positive test results do not imply infectivity.

**Table 2:** Spray‐drying virus inactivation studies

Virus	Nucleic acid	Envelope	Thermal resistance	Virus inactivation	Reference
Porcine reproductive and respiratory syndrome virus	ssRNA	Yes	Low	1 × 10^4.0^	Polo et al. (2005)
Aujezsky disease virus	ssDNA	Yes	Medium	1 × 10^5.3^	Polo et al. (2005)
Swine vesicular disease virus	ssRNA	No	High	1 × 10^6.0^	Pujols et al. (2007)
Porcine epidemic diarrhoea virus	ssRNA	Yes	Low to medium	>1 × 10^5.2^	Pujols and Segalés (2014)
Porcine epidemic diarrhoea virus	ssRNA	Yes	Low to medium	>1 × 10^3.6^	Gerber et al. (2014)
African swine fever virus	dsDNA	Yes	High	1 × 10^4.1^	Blázquez, Pujols, et al. (2018)

Outlet temperature >80ºC.

**Table 3:** Spray‐drying bacteria inactivation studies

Bacteria	Bacteria inactivation	Reference
*Escherichia coli* K88	>1 × 10^7.0^	Blázquez, Rodríguez, Rodenas, et al. (2018)
*E.* *coli* K99	>1 × 10^7.0^	Blázquez, Rodríguez, Rodenas, et al. (2018)
*Salmonella typhimurium*	>1 × 10^9.0^	Blázquez, Rodríguez, Ródenas, et al. (2018)
*Salmonella choleraesuis*	>1 × 10^10.0^	Blázquez, Rodríguez, Ródenas, et al. (2018)

**Table 4:** Summary feeding studies with commercial spray‐dried porcine plasma

Virus	PCR–positive DNA copies	Inclusion level (%)	Feeding duration (day)	Results	Reference
Porcine circovirus‐2	2.47 × 10^5.0^	8	45	Not infective	Pujols et al. (2008)
Porcine circovirus‐2	1 × 10^6.7^	4	42	Not infective	Shen et al. (2011)
Porcine circovirus‐2	7.56 × 10^5.0^	8	32	Not infective	Pujols et al. (2011)
Hepatitis E virus	PCR Pos.	8	28	Not infective	Pujols et al. (2014)
Porcine epidemic diarrhoea virus	PCR Pos.	5	14	Not infective	Campbell, Crenshaw, Polo, Saltzman, and Kesl (2014)
Porcine epidemic diarrhoea virus	PCR Pos.	3–8	7–14	Not infective	Crenshaw, Campbell, et al. (2014)
Porcine reproductive and respiratory syndrome virus	PCR Pos.	3–8	7–21	Not infective	Crenshaw, Pujols, et al. (2014)

This information confirms that the manufacturing process is robust and that SDPP is a safe feed ingredient. The FDA's review of production records and test results of retained samples did not detect infective PEDV or identify a breach in GMP's. There is no evidence to support the suggestion that a breach in GMP's resulted in PEDV contamination of the lot of SDPP being investigated (Aubry et al., 2017).

### 2.4 How was the SDPP sample collected by the CFIA contaminated with infective PEDV?

It is not possible to definitively identify where PEDV contamination of the SDPP sample collected by CFIA occurred.

Testing of SDPP samples resulted in the following observations:

- The FDA pig bioassays did not detect infective PEDV in the manufacturers retained sample from the lot of SDPP investigated by CFIA.
- The sample of SDPP that CFIA collected at the feed mill supplying nursery feed to the Index case was tested in a pig bioassay and was contaminated with infective PEDV.

Independent trials demonstrate that, depending on storage temperatures, PEDV inoculated on SDAP does not survive over 1–3 weeks (Dee et al., 2015; Pujols & Segalés, 2014; Trudeau, Verma, Uriolla, et al., 2017). The SDPP investigated by CFIA was produced over 10 weeks before the Index case and over 13 weeks before CFIA collected their sample (NASDBPP, 2014b). These data support the hypothesis that PEDV contamination of the SDPP sample collected by CFIA occurred after the product left control of the manufacturer during transport or during storage and use at the feed manufacturer.

Following the report of the Index case, all bags of the remaining SDPP inventory (at the feed manufacturer) were sampled multiple times, initially by Plant QA personnel and then by OMAFRA officials before CFIA collected the samples used in the bioassay (MacDougald, 2014b). If PEDV virus was already present in eastern Canada prior to the Index case, and if feed biosecurity protocols were not as rigorous as those in place today, environmental contamination (feed manufacturing facility or equipment) and multiple sampling of the same bag(s) creates the potential for contamination of the SDPP sample CFIA collected and examined in their bioassay.

### 2.5 Bradford‐Hill criterion: association versus causation

It is not necessary to comply with all Bradford Hill criterion to determine causation; however, causation becomes more unlikely as non‐compliance increases. There are several Bradford Hill criteria that the Canadian epidemiology does not comply with.

#### 2.5.1 Consistency of findings

The Canadian epidemiology focused on a specific PEDV outbreak during a limited period in a specific geographic location and linked to a specific feed manufacturer and one production lot of SDPP (Aubry et al., 2017; Pasma et al., 2016; Perri et al., 2018, 2019). In contrast, the conclusions of the Canadian investigation are not consistent with the results of other PEDV outbreak investigations, numerous feeding trials or extensive commercial use of SDPP. For example, epidemiologists were not able to link the PEDV outbreak in Japan with feeding US sourced SDPP (Sasaki et al., 2016). Neumann, Ackerman, Troxel, and Moser (2014) investigated PEDV outbreaks in the Midwest United States and reported that ingredients, including SDPP, had negligible to very low association with the outbreak. Significant volume of US sourced SDPP PCR positive for PEDV was exported to Brazil and Western Canada, enough to feed 3.5 and 4 million pigs, respectively, and the regions remained free of PEDV (Crenshaw, Campbell, et al., 2014; Crenshaw, Pujols, et al., 2014). Numerous review papers have consistently documented increased growth and improved pig health associated with feeding SDPP, with no report of spreading disease (Coffey & Cromwell, 2001; Dewey et al., 2006; Pérez‐Bosque et al., 2016; Remus et al., 2013; Torrallardona, 2010; van Dijk et al., 2001). Conclusions from the 2014 Canadian PEDV investigation that SDPP was responsible for the spread of PEDV are not consistent with numerous other reports. These observations support the hypothesis that other risk factors such as transportation, animal movement or another source of feed contamination contributed to the PEDV outbreak and led to the contaminated SDPP sample collected by CFIA.

#### 2.5.2 Temporal sequence of association

If PEDV was present in the region prior to the Index case and because it was not possible to trace animal and truck movement, then it is not possible to confirm if the Index case was the result of exposure to infective PEDV from the environment or from feed containing the suspected SDPP.

#### 2.5.3 Biological gradient

Results of a case‐controlled study of the Canadian PEDV outbreak conflicted with observation in the initial epidemiological investigation suggesting that increased SDPP inclusion rate resulted in an increase in disease rate. Aubry et al. (2017) reported an increased attack rate associated with increased inclusion rate of SDPP. However, in a case‐controlled study Perri et al. (2018) reported that the dose‐response could not be confirmed.

#### 2.5.4 Experiment

**Figure 4:**
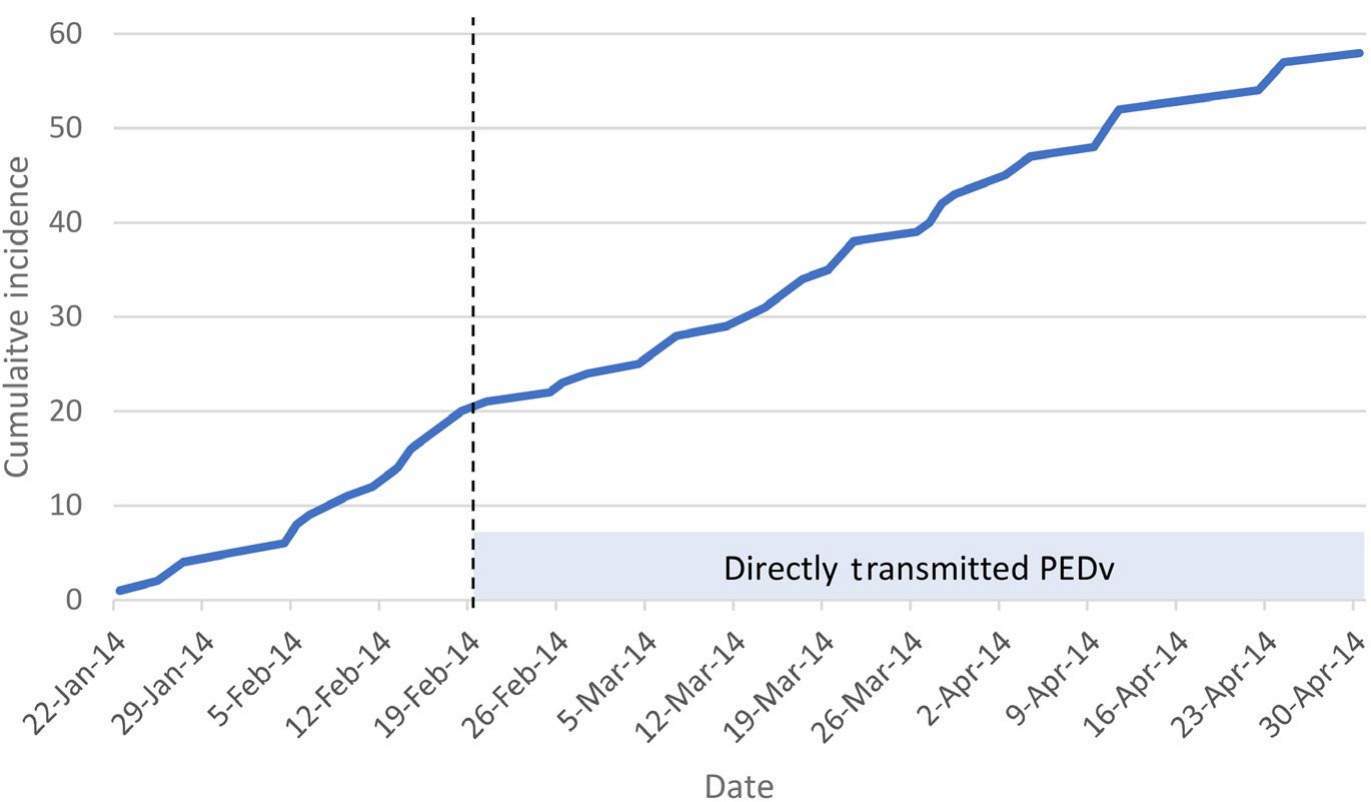
Cumulative incidence data for the Ontario porcine epidemic diarrhoea virus outbreak in 2014. Cases occurring prior to 20 February 2014 (dashed line) are assumed to be the result of a point source exposure through swine feed with cases occurring after 20 February 2014 being the result of direct farm‐to‐farm transmission (adapted from Greer et al., 2017)

Withdrawal of the SDPP containing feed did not alter the rate of new PEDV outbreaks. Greer, Spence, and Gardner (2017) reported the cumulative incidence for the Ontario PEDV outbreaks before and after the withdrawal of SDPP containing feed from the market. There was no change in increase in cumulative incidence rate after withdrawal of the SDPP‐containing feed (Figure 4).

### 2.6 Summary

Many important observations were not included in the epidemiological investigation of the 2014 PEDV outbreak in Canada.

- The timeline associated with PEDV‐positive environmental contamination and the reported Index case was not considered.
- Inadequately cleaned trucks returning to Canada after delivering pigs to US abattoirs was not considered as a source of PEDV introduction in eastern Canada.
- Animal and truck traffic between common sites (including the contaminated Quebec abattoir or assembly yard) and the PEDV‐infected farms were not considered.
- The possibility that imported non‐animal feed ingredients contaminated with PEDV, such as soybean meal, could have introduced PEDV into eastern Canada was not investigated.
- Results of the FDA investigation of the production records and retained samples of the implicated lot of SDPP were not considered.

If these observations were included the conclusions may have changed. For example:

- It is possible that PEDV was present in Canada prior to the Index case.
- It is possible that minimal cleaning of trucks returning from US slaughter plants or PEDV‐contaminated imported non‐animal feed ingredients resulted in the introduction of PEDV into Canada leading to contamination of the Quebec abattoir and the Ontario assembly yard.
- It is possible that contact with the contaminated Ontario assembly yard contributed to the spread of PEDV among many of the initial PEDV‐infected farms.
- There is no support for the suggestion that a breach in good manufacturing practices was responsible for the infective PEDV CFIA reported on the SDPP sample they collected.
- If PEDV was present in Ontario prior to the Index case, multiple sampling of the remaining suspected SDPP at the feed manufacturing site could have contaminated the sample of SDPP tested by the CFIA.

In retrospect, it is not possible to definitively identify the source of PEDV introduced into Canada. It is not possible to definitively determine the source of PEDV contamination in the sample of SDPP tested by CFIA. It will not be possible to definitively determine if truck movement including feed delivery or animal movement was involved in the spread of PEDV. It will not be possible to definitively determine if imported non‐animal feed ingredients introduced PEDV into eastern Canada. However, if the risk factors for the spread of PEDV had been better understood at the time of the initial epidemiology, it is possible that additional data would have been collected during the investigations and the conclusions could have been different.

Both spray‐dried porcine and bovine plasma are important feed ingredients. When included in the diet, SDAP improves growth performance and health of animals. It is important that the SDAP manufacturing process include validated inactivation steps incorporated into the GMP's. The production facility should be designed to prevent cross contamination. When produced properly, SDPP and SDBP are safe effective feed ingredients.

## CONFLICT OF INTEREST

Louis Russell and Javier Polo are employed at APC, LLC, 2425 SE Oak Tree Ct., Ankeny, Iowa, USA, a company that manufactures spray‐dried animal plasma. David Meeker is employed by the North American Renderers Association (NARA). APC, LLC is a member of the NARA.

## ETHICAL APPROVAL

The authors confirm that the ethical policies of the journal, as noted on the journal's author guidelines page, have been adhered to. No ethical approval was required as this is a review article with no original research data.
